# Interference of Competing Beneficial Mutations on Recombining Chromosomes

**DOI:** 10.3390/biology15171561

**Published:** 2026-09-07

**Authors:** Wolfgang Stephan, Stefan Laurent

**Affiliations:** 1 Faculty of Biology, Ludwig-Maximilian University of Munich, D-82152 Planegg-Martinsried, Germany; 2 Department of Comparative Development and Genetics, Max Planck Institute for Plant Breeding Research, D-50829 Cologne, Germany; stefan.laurent@biontech.de

**Keywords:** population genetics, interfering beneficial mutations, fixation times, selective sweeps

## Abstract

Finding signatures of selective sweeps in genomes is a major goal of current population genomics, as it allows estimating the rate of beneficial mutations going to fixation and identifying the genes involved in selection. Models of recurrent selective sweeps traditionally assume that in chromosomal regions of normal recombination rates at most one beneficial allele is on the way to fixation. We review and extend here the theoretical studies on interference between closely linked beneficial mutations suggesting that this assumption may be violated. We show that interference between beneficial mutations may lead to substantially increased fixation times even in chromosomal regions of normal recombination rates. Furthermore, we discuss how interference can be detected in population genomic studies by analyzing genetic footprints of selective sweeps, and search for empirical evidence of interference in published datasets.

## Simple Summary

The traditional view has been that in genomic regions with normal recombination only a single beneficial mutation spreads through a population at a time and becomes fixed. In this study, we challenge this view by demonstrating that interference between beneficial mutations can significantly prolong fixation time. We extend the analysis of a mathematical model to estimate the time until two simultaneously occurring beneficial mutations recombine and jointly go to fixation. Furthermore, we discuss how interference may be detected in data on DNA sequence variation by linking our theoretical predictions to genomic signatures of selective sweeps, a well-studied field in current population genetics. Finally, applications to *Drosophila* and deer mice datasets lead to useful insights into the detectability of interference.

## 1. Introduction

The speed of beneficial mutations on their way to fixation in natural populations is a fundamental topic in population genetics. Knowing the time for a single beneficial allele to spread through a natural population and going to fixation is essential for understanding evolution. Fixation time is also an important concept for analyzing the dynamics of multiple interacting mutations or alleles. However, the spread of two or more beneficial mutations that arise and interact during their fixation process is much less investigated.

For two interacting mutations, Otto and Barton [1] have studied the case that the second mutation is less beneficial than the first one. In contrast, Cuthbertson et al. [2] and Bossert and Pfaffelhuber [3] considered the scenario that the second mutation is fitter than the first one. Furthermore, they assumed that there is a chance that the first and second mutants recombine such that the recombinant type has the highest fitness and eventually fixes. This model of competing beneficial mutations has also been studied in a series of other papers, including [4,5,6]. Barton [4] and Yu and Etheridge [5] were interested in the probability of fixation, and Christiansen et al. [6] investigated the waiting time to the formation of a double mutant. Here we will focus on the fixation time of two competing beneficial mutations.

We consider the analysis of the fixation time of interfering beneficial mutations in conjunction with the theory of selective sweeps and show that sweeps can be used to detect interference in whole genome sequence data. Although beneficial mutations are a comparatively small fraction of all new mutations, some of them may reach fixation and are thus important in evolution. If the fitness effects of these beneficial mutations are sufficiently strong, they may cause selective sweeps, i.e., localized reductions in genetic variation along recombining genomes [7].

Finding signatures of selective sweeps in genomes is a major goal of current population genetics, as it allows estimating the rate of beneficial mutations going to fixation and identifying the genes involved in selection. Models of recurrent selective sweeps traditionally assume that in chromosomal regions of normal recombination rates at most one beneficial allele is on the way to fixation [8,9]. However, later studies showed (mostly by computer simulations) that for a high rate of selected substitutions and a low rate of recombination this assumption may be violated. Kim and Stephan [10] found that interference between linked beneficial mutations causes a reduction in their fixation probabilities; furthermore, the hitchhiking effect on linked neutral variation for a given substitution slightly decreases. They concluded that this effect is significant only in chromosomal regions of relatively low recombination rates and that previous assumptions on recurrent selection are still largely valid in regions of normal recombination. Simulations by Chevin et al. [11], however, cast doubts on these conclusions. They studied variation at neutral sites located between two closely linked selected loci when both beneficial mutations reach fixation, assuming normal recombination rates. They found that the beneficial mutations may interfere on their way to fixation, such that nucleotide diversity is less reduced than in the case of a single sweep of the same selection strength, and most importantly, they observed that for some parameter values interference may induce an excess of intermediate-frequency variants relative to neutrality.

However, a problem with the work of Chevin et al. [11] is that it is very difficult, if not impossible, to study their model analytically. For this reason, Stephan [12] investigated the effect of interference of two competing beneficial mutations analytically and then compared the results to the simulations of Chevin et al. [11]. He concentrated on the scenario that led to an excess of intermediate-frequency variants. He assumed that, while a highly beneficial mutation spreads in a natural population, a second beneficial mutation arises before the first one has fixed. Furthermore, he envisioned that the first mutation is less fit than the second one and that recombination may occur between the chromosomes carrying the mutations. Under these conditions a haplotype may be formed that is fitter than the two individual mutations and therefore eventually fixes. To model this process for a population of finite size, Bossert and Pfaffelhuber [3] had used stochastic differential equations and calculated the fixation time of a recombinant haplotype under the assumption that it fixes. In contrast, Stephan [12] used a deterministic model based on ordinary differential equations (ODEs) that has the inherent property that the recombinant haplotype is formed and eventually fixes.

The present paper is structured as follows. In Section 2 we describe and update the theoretical work of Stephan [12]. In Section 2.1 the basic ODEs for allele frequency changes are formulated, while in Section 2.2 the analytical results for the fixation times are shown. Two cases are distinguished in the latter section: the repulsion case in which the second mutation arises on a chromosome that is different from the first one, and the association case in which the second mutation appears on the same chromosome as the first one. Then, in Section 3 we describe the parallel of fixation times and selective sweeps by comparing the simulation data of Chevin et al. [11] to the length of fixation times derived in Section 2.2. Thus, we argue that analysis of the genomic footprints of selective sweeps can be used to investigate interference between competing beneficial mutations. Finally, in Section 4 we use examples of published DNA polymorphism data to search for empirical evidence of interference in recombining genomic regions.

## 2. Theory

### 2.1. Model

We consider a two-locus model, with alleles A and a at locus 1 and B and b at locus 2, respectively. The upper-case letters denote beneficial alleles with selection coefficients $s_{2}$ at locus 1 and $s_{3}$ at locus 2, whereas the alleles with lower-case letters are assumed to be neutral (wildtype). This model has four haplotypes AB, Ab, aB, and ab, with frequencies given by the variables $X_{1},\;X_{2},\;X_{3\;}$ and $X_{4}$ (which add up to 1). Assuming additive selection, their relative fitnesses are $1+s_{1}$, $1+s_{2}$, $1+s_{3\;}$ and 1, respectively, where $s_{1}=s_{2}+s_{3}$. Note that we assume additive fitness and neglect epistatic fitness interactions between the beneficial mutations. Recombination between locus 1 and locus 2 occurs at rate r. Since we are interested in closely linked loci, we assume that $r\ll s_{i}\ll 1$ for i = 2, 3. In our deterministic setting (without genetic drift) the ODEs for the time change in the variables $X_{i}$ are obtained by adding the change due to selection and the change due to recombination ([13], Ch. 2):

(1) $$\frac{dX_{1}}{dt}=X_{1}\left(s_{1}-\sum_{j=1}^{3}s_{j}X_{j}\right)-r\left(X_{1}X_{4}-X_{2}X_{3}\right),\;\frac{dX_{2}}{dt}=X_{2}\left(s_{2}-\sum_{j=1}^{3}s_{j}X_{j}\right)+r\left(X_{1}X_{4}-X_{2}X_{3}\right),\;\frac{dX_{3}}{dt}=X_{3}\left(s_{3}-\sum_{j=1}^{3}s_{j}X_{j}\right)+r\left(X_{1}X_{4}-X_{2}X_{3}\right),\;\frac{dX_{4}}{dt}=X_{4}\left(-\sum_{j=1}^{3}s_{j}X_{j}\right)-r\left(X_{1}X_{4}-X_{2}X_{3}\right),$$

where t measures time in generations.

For r>0, which we assume throughout this paper, an exact solution of this system of ODEs is not known. An approximate solution can be obtained using nonnormalized variables $Y_{i}$ (*i* = 1,… 4) (see [14] for mutation-selection and [15] for recombination-selection equations). These are related to the original variables of Equation (1) as:

(2) $$X_{i}=\frac{Y_{i}}{\sum_{j=1}^{4}Y_{j}}\;.$$

As shown in [12], approximate formulae for the nonnormalized variables can be derived in the case that both mutations A and B arise on background ab (so that $X_{1}\left(0\right)=0).$ In the following we refer to this situation as repulsion case. Similar expressions can be obtained for the so-called association case, in which we assume that $X_{1}\left(0\right)>0$ and $X_{3}\left(0\right)=0.\;$ The terms ’repulsion’ and ’association’ (or ’coupling’) describe the arrangement of two types of alleles (such as mutation and wildtype) at two loci on a chromosome, as it is best known from the literature on linkage disequilibrium ([16], Ch. 10). Their biological meaning is that mutations arise on different versus the same chromosomal background. Using (2) approximate expressions for the original variables $X_{i}$ may be obtained from the $Y_{i}$.

### 2.2. Fixation Times

To derive the fixation time of two competing beneficial mutations, we use the approximate formulas for $Y_{i}$ [12]. We call an allele or haplotype fixed when it reaches frequency 1−δ  and denote this time T. Thus, T measures the time from some initial frequency at t=0  to 1−δ. The initial frequency may be the frequency of a newly arising mutation. In a haploid population of size N, which we consider here, this initial frequency is given by 1/N. This is the case when the mutant B appears in association with A; i.e., on an Ab haplotype. In the repulsion case, however, we are interested in the fixation of the double mutant AB whose initial frequency is $X_{1}\left(0\right)=0,\;$ as AB arises during the fixation process due to recombination. In both cases, the fixation time of AB is found by solving the equation

(3) $$X_{1}\left(T\right)=1-\delta ,$$

where δ is a small number. *T*, of course, may be different in both cases and will be denoted by $T^{a}$ in the association and by $T^{r}$ in the repulsion case.

#### 2.2.1. Fixation Time for the Repulsion Case

We begin by expressing Equation (3) in terms of nonnormalized variables and obtain

(4) $$X_{1}\left(T\right)={\left(1+\sum_{i=2}^{4}\frac{Y_{i}(T)}{Y_{1}(T)}\right)}^{-1}=1-\delta .\;$$

Equation (4) follows directly from Equation (2) by pulling out $Y_{1\;}$ in the denominator and then canceling it against $Y_{1\;}$ in the numerator. Because the term Y4Y1 can be neglected in the sum, we obtain in both repulsion and association cases the equation

(5) $$\delta \approx \frac{Y_{2}\left(T\right)}{Y_{1}\left(T\right)}+\frac{Y_{3}\left(T\right)}{Y_{1}\left(T\right)}.$$

In our model we generally require that for Equation (1) $X_{2}\left(0\right){>X}_{3}\left(0\right)$ and $s_{3}>s_{2}$. Furthermore, we assume that $T^{r}\;$ is sufficiently large such that the second mutation is eventually dominating the first one; i.e., $X_{2}(0)e^{s_{2}T^{r}}\ll X_{3}(0)e^{s_{3}T^{r}}$, and population size is large $\left(N>10^{5}\right)\;$ such that selection is strong $(Ns_{i}>100).\;$ Under these assumptions we find [12]

(6) $$-\frac{\delta X_{2}(0)ln\left(X_{2}\left(0\right)\right)}{s_{2}\left(1-X_{2}\left(0\right)\right)}\approx e^{-s_{2}T^{r}}\left(\frac{1}{r}-\left(\frac{s_{3}ln\left(X_{2}\left(0\right)\right)}{s_{2}\left(s_{3}-s_{2}\right)}-\frac{ln\left(X_{3}\left(0\right)\right)}{s_{3}-s_{2}}\right)\right).$$

Finally, we introduce population size N into this equation by writing $\delta =\frac{1}{N}{,\;X}_{3}\left(0\right)=\frac{1\;}{N}$ and $X_{2}\left(0\right)=\frac{x_{20}}{N}$, where $x_{20}\;$ is the number of A alleles at t=0; in other words, the scaled frequency of A at the time when B is introduced. Then solving the equation for $T^{r}\;$ yields

(7) $$T^{r}\approx \frac{1}{s_{2}}\left[2ln\left(N\right)-ln\left(\frac{x_{20}}{s_{2}}\right)-ln\left(\frac{ln\left(\frac{N}{x_{20}}\right)}{1-\frac{x_{20}}{N}}\right)+ln\left(\frac{1}{r}-\left(\frac{ln\left(N\right)}{s_{3}-s_{2}}-\frac{s_{3}}{s_{2}}\frac{ln\left(\frac{N}{x_{20}}\right)}{s_{3}-s_{2}}\right)\right)\right].$$

The first term on the right-hand side of Equation (7) equals the fixation time of a new allele starting at frequency $\frac{1}{N\;}\;$ and ending at$\;1-\frac{1}{N\;}$, driven by positive directional selection with selection coefficient$\;s_{2}$. This term also appears in the result of Bossert and Pfaffelhuber [3]. The denominator $s_{2\;}$ can be explained as follows. To go to fixation, the successful recombinant AB with fitness $1+s_{2}+s_{3\;}$ has to compete against the—at the time—dominant aB type with fitness $1+s_{3\;},\;$ having a fitness advantage $s_{2}.$

Furthermore, as shown in Appendix A, the term $\frac{ln\left(N\right)}{s_{3}-s_{2}}-\frac{s_{3}}{s_{2}}\frac{ln\left(\frac{N}{x_{20}}\right)}{s_{3}-s_{2}}$ is identical to $\frac{1}{s_{3}-s_{2}}\left(1-\frac{s_{3}}{s_{2}}\psi \right)ln\left(N\right).\;$ The latter term also appears in [3]. It describes the mean time from the occurrence of the second mutation to its establishment in the population at some low level, such that recombination between Ab and aB can occur (see the discussion around Equation (2.6) in [3]). Note that the denominators of the term $\frac{ln\left(N\right)}{s_{3}-s_{2}}-\frac{s_{3}}{s_{2}}\frac{ln\left(\frac{N}{x_{20}}\right)}{s_{3}-s_{2}}\;$ contain the difference in the selection coefficients between the second and first mutation indicating that the two mutations compete against each other.

In the interesting parameter range of small r values such that $\frac{1}{r}\gg \frac{ln\left(N\right)}{s_{3}-s_{2}}-\frac{s_{3}}{s_{2}}\frac{ln\left(\frac{N}{x_{20}}\right)}{s_{3}-s_{2}}$, we may approximate Equation (7) as

(8) $$T^{r}\approx \frac{1}{s_{2}}\left[2ln\left(N\right)-ln\left(\frac{r}{s_{2}}\frac{x_{20}}{1-\frac{x_{20}}{N}}ln\left(\frac{N}{x_{20}}\right)\right)\right].$$

Thus, unless r  is very small, the second term in Equation (8) is negative such that $T^{r}$ is smaller than $\frac{2ln\left(N\right)}{s_{2}}$. This is not surprising as we are dealing here with an equation describing continuous input of new AB alleles due to recombination, similar to the case of fixation under continuous mutation pressure and positive directional selection. Based on Equation (8) we obtain a relatively simple formula for a threshold value of r

(9) $$r_{c}=\frac{s_{2}}{x_{20}}\frac{1-\frac{x_{20}}{N}}{ln\left(\frac{N}{x_{20}}\right)}\;.$$

Therefore, Equation (8) can be written as

(10) $$T^{r}\approx \frac{1}{s_{2}}\left[2ln\left(N\right)-ln\left(\frac{r}{r_{c}}\right)\right].$$

Thus, for r> $r_{c}\;$ fixations occur faster than $\frac{2ln\left(N\right)}{s_{2}}$, whereas for $r<r_{c}\;$ the second term in Equation (8) turns positive, such that the fixation time $T^{r}$ becomes larger than $\frac{2ln\left(N\right)}{s_{2}}$, meaning that the input of recombinants slows down.

**Figure 1:**
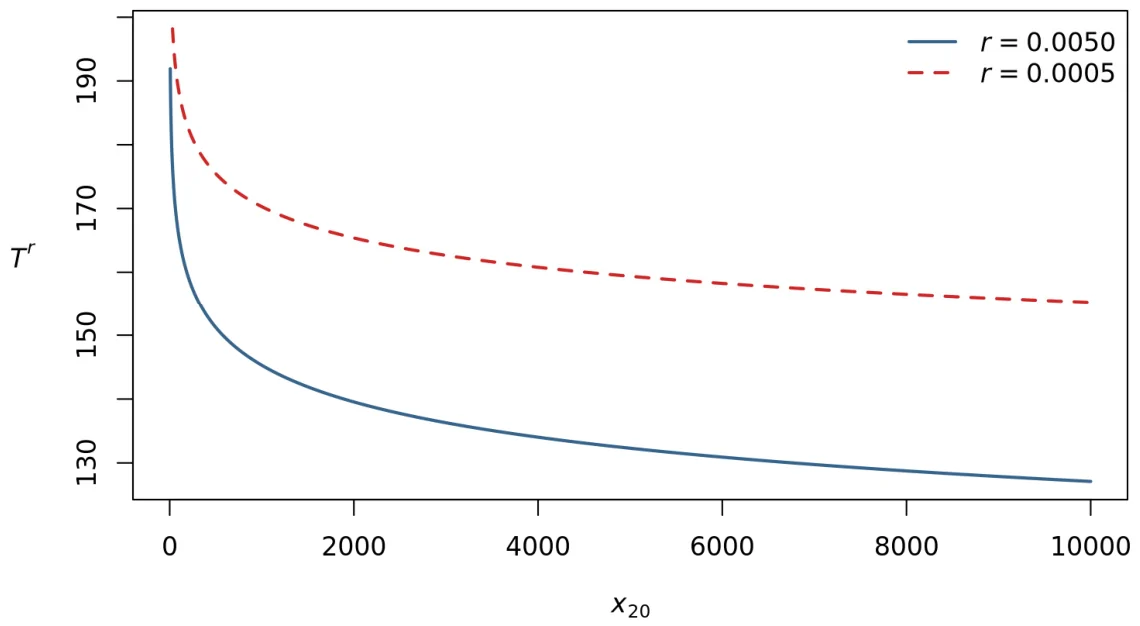
Fixation time $T^{r}$ versus number of *A* alleles, $x_{20},\;$at the introduction of mutation B. $T^{r}$ is the fixation time in the repulsion case from Equation (7) measured in generations and $x_{20}$ is the number of A alleles at the introduction of mutation B. The parameter values are: $N=20,000,\;s_{2}=0.1,\;s_{3}=0.2,\;and\;r=0.005\;or\;0.0005.$

Finally, we visualize the fixation time $T^{r}$ as a function of$\;x_{20}\;$using Equation (7) for fixed values of $N,\;s_{2},\;and\;s_{3}$ and also show the effect of recombination (Figure 1). The fixation time is plotted for two recombination rates, r=0.005  and 0.0005, such that the variable $x_{20}$ satisfies the condition $\frac{x_{20}}{1-\frac{x_{20}}{N}}ln\left(\frac{N}{x_{20}}\right)>\frac{s_{2}}{r}.$ Both curves have their maximum $\frac{2ln\left(N\right)}{s_{2}}\;$ at a low value of $x_{20\;}\;$and decrease for increasing introduction frequencies. This decay is most pronounced for small $x_{20}\;$ values. As expected, a lower recombination rate leads to a longer fixation time.

Note that population size in Figure 1 (and also in Table 1) is chosen as N=20,000. This is lower than the value $N>10^{5}$ suggested above to guarantee the validity of the approximations in the repulsion case. The lower value of N was used by Chevin et al. [11]. However, the lower value of N did not cause any discrepancies in the approximations (see the discussion in the first paragraph of Section 4 in [12]).

#### 2.2.2. Fixation Time for the Association Case and Average Fixation Time

Evaluating Equation (5) for the association case, we find

(11) $$\delta {(1-r(1-X_{2}\left(0\right))I}_{1}\left(T^{a}\right))\approx \;\frac{1}{X_{1}\left(0\right)e^{s_{3}T^{a}}}\left(X_{2}\left(0\right)+rX_{1}\left(0\right)(1-X_{2}\left(0\right))I_{2}\left(T^{a}\right)\right)+\frac{r(1-X_{2}\left(0\right))I_{3}\left(T^{a}\right)}{e^{s_{2}T^{a}}}\;,$$

where the integrals are defined as

(12) $$I_{1}\left(T^{a}\right)\approx \int_{0}^{T^{a}}\frac{1}{X_{4}\left(0\right)+X_{1}\left(0\right)e^{s_{1}\tau }+X_{2}\left(0\right)e^{s_{2}\tau }}d\tau ,\;I_{2}\left(T^{a}\right)\approx \int_{0}^{T^{a}}\frac{e^{s_{3}\tau }}{X_{4}\left(0\right)+X_{1}\left(0\right)e^{s_{1}\tau }+X_{2}\left(0\right)e^{s_{2}\tau }}d\tau ,\;I_{3}(T^{a})\approx \int_{0}^{T^{a}}\frac{e^{s_{2}\tau }}{X_{4}\left(0\right)+X_{1}\left(0\right)e^{s_{1}\tau }+X_{2}\left(0\right)e^{s_{2}\tau }}d\tau .$$

Precise approximations that are valid for the entire interval $0{\leq X}_{2}\left(0\right)\leq 0.5$ as in the repulsion case ([12], Equation (7)) could not be obtained for these integrals. Instead, we end up with the following equation for $T^{a}$

(13) $$\delta {{(1-r(1-X_{2}\left(0\right))I}_{1}\left(T^{a}\right))e}^{s_{3}T^{a}}-r\left(1-X_{2}\left(0\right)\right){I_{3}\left(T^{a}\right)e}^{\left(s_{3}-s_{2}\right)T^{a}}\;-\left(\frac{X_{2}\left(0\right)}{X_{1}\left(0\right)}+r(1-X_{2}\left(0\right))I_{2}\left(T^{a}\right)\right)\approx 0.$$

In general, this is a transcendental equation that cannot be solved analytically for $T^{a}$. For the numerical work Maxima or Mathematica may be used. For the parameter values used in Table 1, we find that $T^{a}\;$ is substantially shorter than $T^{r}$ and relatively constant for increasing $X_{2}\left(0\right)$. Furthermore, we note that $T^{a}$ decreases for increasing $X_{2}\left(0\right)$ as in the repulsion case (although to a lesser extent), such that it eventually converges to an asymptotic value. The asymptote can be found by inserting a large value for $X_{2}\left(0\right)\;$ into Equation (13). This leads to

(14) $$T^{a}\approx \frac{2}{s_{3}}ln\left(N\right)\;.$$

For the given parameter values of Figure 1 and Table 1, the asymptote calculated from Equation (14) is 99.03, which is slightly above the numerical result (≈97.0) obtained from Equation (13). Here the denominator $s_{3}\;$differs from that in Equation (7) because on the way to fixation the successful haplotype *AB* with fitness $1+s_{2}+s_{3\;}$ has to compete against the—at the time—dominant *Ab* type with fitness $1+s_{2\;},\;$having a fitness advantage $s_{3}.$

Since the introduction of the second mutation into the population depends on the frequency of A, we may average the times to fixation for both the repulsion and association cases. The average fixation time T is therefore obtained as

(15) $$T=\left(1-X_{2}\left(0\right)\right)T^{r}+X_{2}\left(0\right)T^{a}.$$

T exhibits the same features as $T^{r}$ (Table 1): a steep decay for small $X_{2}\left(0\right)\;$ and leveling off for larger introduction frequencies. For small values of $X_{2}\left(0\right),$ interference between beneficial mutations is therefore strongest.

## 3. Detecting Interference Between Competing Beneficial Mutations Using Selective Sweeps

How can interference between competing beneficial mutations be detected? It may be hard to estimate the fixation time of closely linked beneficial mutations using a computational approach, such as based on coalescent theory. However, the effect of these mutations on linked neutral variation in their neighborhood may relatively easily be detected. Using full-forward simulations, Kim and Stephan [10] found that interference between linked beneficial alleles causes a reduction in their fixation probabilities. Furthermore, investigating a model with two selected sites and one neutral locus (between the selected ones) they noticed that an excess of neutral variation may occur when the neutral site was located between the two selected ones. Similar results were obtained with one neutral site and many selected loci.

A more detailed analysis of a similar model with two selected sites and three neutral ones was performed by Chevin et al. [11]. These authors studied genetic variation at the three neutral sites located between two closely linked, selected loci, using Monte Carlo simulations of a Wright-Fisher model ([13], Ch. 3). Of the three neutral loci, the left one is close to the selected locus 1, the right one near locus 2, while the third neutral locus is in the middle between loci 1 and 2. Typical parameter values used in the simulations (including values of the recombination rate r between the two selected sites and the introduction frequency $X_{2}\left(0\right)\;$ of B) are given in the captions to Figure 1 and Table 1.

Chevin et al. [11] report that the two loci close to the selected ones show typical hitchhiking effects [7]; i.e., variation is reduced relative to the neutral standard level such that stronger selection acting at locus 2 $\left(s_{3}>s_{2}\right)\;$ leads to a greater reduction than at the neutral site near locus 1. These observations are summarized in Table 1. Furthermore, for $X_{2}\left(0\right)<0.1\;$ variation at the neutral locus in the middle between the two selected loci is considerably larger than that expected for selection at a single locus with selection coefficient $s_{2}\;$or $s_{3}$ [7]. This excess of neutral variation between two interfering selected mutations arises because the effect of each allele in reducing linked variation is diminished due to interference. A similar excess of neutral variation between two closely linked selected sites was also observed by Kim and Stephan [10].

Furthermore, as shown in Table 1, increasing $X_{2}\left(0\right)\;$ generally leads to stronger hitchhiking effects such that levels of neutral variation decrease with $X_{2}\left(0\right)$. This can be clearly observed at the neutral locus close to locus 1, whereas at the neutral locus close to the stronger selected site the decrease in neutral variation is less pronounced. At the neutral locus in the middle there is also a strong decay of variation with increasing levels of $X_{2}\left(0\right)$. The effect of $X_{2}\left(0\right)$ on hitchhiking is likely due to the interference of the two selected mutations. The longer they compete with each other on their way to fixation, the stronger their interference and the weaker their hitchhiking effect.

A statistic traditionally used for describing effects of selective sweeps is Tajima’s [17] D. Chevin et al. [11] also used it for describing genomic footprints of competing selective sweeps. In Table 1 the D values for the neutral locus in the middle between locus 1 and 2 are shown. All D values at the other two loci are negative as expected from the theory of genetic hitchhiking. A negative D is observed when an allele has either a lower or higher frequency than expected by the neutral theory. Positive values of D indicate that alleles are at intermediate frequencies. Interestingly, Chevin et al. [11] observed strongly positive D values for $X_{2}\left(0\right)=0.007,\;0.024,\;$ and 0.077, whereas D is around zero or negative for larger $X_{2}\left(0\right).$ A plausible hypothesis proposed by Bossert and Pfaffelhuber [3] is that positive D may be observed when a haplotype structure arises in the genome through recombination between different haplotypes consisting of multiple polymorphic loci. Haplotype structures exist in populations only if polymorphisms at individual loci tend to be in intermediate frequency (such that the less frequent variants are not too rare). This may be the case for $X_{2}\left(0\right)=0.007,\;0.024,\;$ and 0.077, but not for the larger $X_{2}\left(0\right)$ values, for which diversity is more heavily reduced (Table 1). An alternative, though related, hypothesis postulates that the dynamics of the two selected mutations (while in repulsion) reaches nonnegligible frequencies at similar times such that recombination may produce haplotypes with the two favorable alleles in coupling [11].

Finally, we compare the characteristic properties of the fixation time T of two competing mutations to the footprints of selective sweeps observed by Chevin et al. [11]. We note that the steep decay of T occurs in the same range of $X_{2}\left(0\right)\;$ as the elevated levels of Tajima’s D (Table 1). For these low values of $X_{2}\left(0\right),$ the interference between the beneficial mutations is strongest.

To conclude this section, by comparing our theoretical results with the simulations of Chevin et al. [11] we have presented evidence that interference between closely linked beneficial mutations may be detected by analyzing footprints of selective sweeps in the genome. In the following, we apply this insight to two published datasets of DNA sequence polymorphisms.

## 4. Searching for Evidence of Interference Between Beneficial Mutations

In Stephan [12], we discussed two cases in which selected substitutions (selected sweeps) occurred in close proximity in the genome. One of them was found at the *polyhomeotic* locus of a European population of *D. melanogaster*. Voigt et al. [18] identified five highly differentiated SNPs in the region between *polyhomeotic proximal* and the gene *CG3835* and showed that the derived European 5-kb haplotype is associated with reduced thermosensitivity of expression, but they did not functionally test each SNP separately. Variation is generally low in the whole *polyhomeotic* region and Tajima’s D is strongly negative, as expected after a sweep. However, there is no evidence that the five beneficial substitutions that likely caused sweeps acted independently in a sequential manner. Instead, they probably migrated jointly from the ancestral population in Africa into the European population and were selected as haplotype block. As a consequence and in agreement with the predictions of this study, an elevated level of Tajima’s D in the fragment containing the five selected substitutions was not detected.

A second possible example of interference between beneficial mutations may be the *Agouti* locus in Nebraska deer mice. The Sand Hills of Nebraska were formed 8000–10,000 years ago and were colonized since about 4000 years by light-colored mice from a dark-colored population (mostly) to the south [19]. To search for evidence of interference at the *Agouti* locus, we re-analyzed the polymorphism data of Pfeifer et al. [19]. These data are available at: https://figshare.com/s/7ece137411ae5cf8ba56?file=8706658 (accessed on 27 March 2026).

The data were collected from 11 locations across a 330 km south–north transect, including six samples from populations off the Sand Hills (266 individuals), in the following called OFF populations, and five samples from populations on the Sand Hills (271 individuals), ON populations. To minimize the number of false positives in the sequence data, variants were subjected to several stringent filter criteria. Population genetic statistics, such as $F_{ST}$ and Tajima’s D (with varying window sizes), were estimated by standard methods mentioned in [19]. To map potential selective sweeps, the composite likelihood ratio (CLR) test was utilized.

Genomic footprints of sweeps may be detected for up to 0.1 N generations ago for randomly mating populations [20]. Given that one year corresponds to about 2–4 generations for deer mice and the (effective) population size N is about 45,000 [19], only relatively young individual selective sweeps may be detected. Evidence for sweeps was found in the large Intron 1 and the adjacent region between Exon 1A and the duplicated reversed copy Exon 1A’ by the CLR test ([19], Figure 6). In Exon 1A a region has been identified by the CLR method containing two closely linked selected sweeps. However, evidence for interference is not found in this region, as Tajima’s D is not elevated ([19], Figure 6).

In the remaining *Agouti* gene (in which significant CLR tests were not found; i.e., the region with genomic positions up to approximately 25,120 kb), evidence for positive selection was detected, using measures of genetic differentiation (in particular the HapFLK method; [21]). Pfeifer et al. [19] found a significant peak of differentiation between ON and OFF Sand Hills populations around the site of an amino acid deletion (called Region 1, coordinates 25,073.746 to 25,078.520 kb), which arose de novo after formation of the Sand Hills [22] and was shown in subsequent field and laboratory experiments to be linked to survival in wild deer mice [23]. Even stronger differentiation between the ON Sand Hills populations and the OFF population to the south was found in Region 2 (coordinates 25,101.801 to 25,111.948 kb), located in Intron 1 near Exon 1. Here, a high peak in the HapFLK profile co-localizes with a region of high linkage disequilibrium [19], suppl. Figure 8, suggesting a recent selective event within Region 2.

In Region 2, fixed differences between ON and OFF populations or variants fixed in the ON sample, but polymorphic in the OFF populations (similar to the *Drosophila* case) were not found. However, we detected eight nucleotide sites at which there was a large frequency difference between the ON and South-OFF variants. $F_{ST}$ at these sites ranged from 0.228 to 0.439. This may suggest that the much larger frequencies in the ON population at these eight sites are due to positive selection. Therefore, these sites may have contributed to the large peak of HapFLK. An even a greater number of large $F_{ST}$ values was also found at other sites in Region 2, at which the frequency of the variant in the South-OFF sample was larger than in the ON sample. However, these frequency differences may be attributed to migration and drift, at least to some extent.

Eight positively selected variants within Region 2 (about 10 kb) may suggest a case of interference. This claim is consistent with the observation that Tajima’s D values around six of these sites are high (the highest across the generally high *D* values of Region 2; see Figure 6 in [19]) and the sites are clustered in two groups of three closely linked sites each. In the first group, the sites are 431 bp apart. Of the three sites two neighboring sites show very similar frequencies in both ON and OFF populations suggesting that they evolved as a haplotype block. In the second group, the sites are 373 bp apart and seem to have evolved independently. The high D values in their immediate neighborhood indicate that the times to fixation of the beneficial variants entering the ON population are longer than expected because of interference [12]. The two clusters of sites are 6.8 kb apart.

In Region 1 the results are less striking. We found four sites with substantial (positive) frequency differences between ON and South-OFF populations and $F_{ST}$ values between 0.157 and 0.230. The four sites are distributed across a 2.7-kb region, and Tajima’s D values are not substantially elevated.

## 5. Discussion

To investigate the interference between competing beneficial mutations we discussed in Section 2 the following population genetic scenario. While a highly beneficial mutation A at locus 1 is spreading in a very large population, a second beneficial mutant B arises at locus 2 before *A* has fixed. Under the assumptions that the fitness of B is greater than that of A and that A- and B-carrying chromosomes can recombine, recombinants AB form and eventually fix. Then we present approximate formulas for the fixation time T of AB under additive fitness of the mutations as a function of $X_{2}\left(0\right),\;$the frequency of A at the introduction of B. The latter parameter turns out to be useful for describing the interference between competing beneficial mutations. Our analysis suggests that the effect of interference between beneficial mutations is most pronounced for small values of $X_{2}\left(0\right).\;$In this parameter range fixation time may increase substantially with decreasing $X_{2}\left(0\right).$ However, for larger values of $X_{2}\left(0\right)\;$fixation time is relatively constant (Figure 1, Table 1).

In our model we assume additive fitnesses; i.e., the relative fitnesses of the mutations A and B are $1+s_{2}$ and $1+s_{3\;}$, respectively, while $s_{1}=s_{2}+s_{3}$ is the relative fitness of the haplotype AB. Thus, as we mention in Section 2.1, we neglect epistatic fitness interactions between the beneficial mutations. Whether this assumption can be viewed as a limitation of our analysis or whether our results can be generalized to the case $s_{1}{\;\neq s}_{2}+s_{3}$ is currently unknown.

For a population genetic model identical to ours, and strong selection, Bossert and Pfaffelhuber [3] used a stochastic approach to calculate the fixation time of a recombinant haplotype under the assumption that it fixes. Of course, the results of the two approaches are not identical. Most importantly, the recombination parameter r does not appear in the equations that Bossert and Pfaffelhuber derived. However, other parts of their formulas match completely with our result (discussed in the two paragraphs below Equation (7)). This suggests that a deterministic approach is valid for the calculation of fixation time when selection is strong (Ns≫1), as also holds for other aspects of the theory of genetic hitchhiking (selective sweeps).

In Section 3 we showed that the behavior of fixation time T as a function of $X_{2}\left(0\right)\;$parallels the effect of interference on the genomic footprint of competing mutations observed in the simulations of Chevin et al. [11]. For small values of $X_{2}\left(0\right)$, these authors found a strongly positive Tajima D, whereas D is around zero or negative for larger $X_{2}\left(0\right)\;$(Table 1). Positive values of D indicate that alleles are in intermediate frequencies. This may be observed when a haplotype structure arises in the genome through recombination between different allelic types consisting of multiple polymorphic loci [3,11].

Finally, we asked in Section 4 whether interference can be encountered in real data; in particular, in genomic regions of normal recombination rates. Note that the recombination rate used by Chevin et al. [11] exceeds the critical rate $r_{c}$ defined in Equation (9). To address this question, we need data on selection coefficients of beneficial mutations and the frequency of selected substitutions, which may come from studies of selective sweeps. Estimates of the average selection coefficient s and the rate ν,  at which beneficial mutations arise and go to fixation (i.e., selective substitutions), are known for some species including *Drosophila melanogaster*. For instance, Jensen et al. [24] analyzed a dataset of genetic variation from the euchromatic part of the genome of a *D. melanogaster* population from Africa, which is—roughly speaking—the recombining portion of chromosomes. They obtained the following estimates: s=0.002, $N=2.5\times 10^{6}\;$and $\nu =4.2\;{\times 10}^{-11}\;$ per generation per nucleotide site. Since under strong selection the mean fixation time for a diploid species such as *D. melanogaster* is $T=\frac{2}{s}ln\left(2N\right)$, where N is the diploid population size, we find that the probability of a second substitution arising on a chromosome during the sojourn of the first one to fixation is $T\nu =6.5\times 10^{-7}$ per nucleotide site. Multiplying Tν with the size of the euchromatic part of a chromosome (in *D. melanogaster* approximately 24 Mb $=2.4\times 10^{7}\;$ base pairs), we find that on average at about 15.6 sites of a chromosome strongly selected substitutions could arise. However, to compete with the first mutation during its sojourn to fixation, they must be sufficiently linked. Another necessary condition for interference to occur is that the second mutation arises as long as the first mutation is at low frequency, say $X_{2}\left(0\right)<0.1\;$ (see Section 3). The sojourn time of the first mutation (with selection coefficient s) in an interval $X_{2}\left(0\right)<0.1$ is approximately $T_{2}=\frac{1}{s}ln\left(2NX_{2}\left(0\right)\right)\approx 6.6\times 10^{3}$ generations, whereas the fixation time is about 1.5 $\times \;10^{4}$ generations. The sojourn time was calculated from Equations (4.25) and (4.41) in Ewens ([13], Ch. 4). These results suggest that beneficial mutations spend a relatively long time at low frequencies before they go on to fixation.

We discussed two cases in which selected substitutions (selected sweeps) occur in close proximity in the genome. One of them was found at the *polyhomeotic* locus of a European population of *D. melanogaster*. Voigt et al. [18] report that five selected substitutions (i.e., nearly fixed variants between Europe and Africa) are located in the 5-kb intergenic region between *polyhomeotic proximal* and the gene *CG3835*. They showed that the derived European 5-kb haplotype is associated with reduced thermosensitivity of expression, but they did not functionally test each SNP separately. Thus, there is no evidence that the five beneficial substitutions that likely caused the sweep acted independently in a sequential manner. Instead, they probably migrated jointly from the ancestral population in Africa into the European population and were selected as haplotype block. As a consequence and in agreement with the predictions of this study, an elevated level of Tajima’s D in the fragment containing the five selected substitutions was not detected.

A second example in the context of interference between beneficial mutations may be the *Agouti* locus in deer mice. Here, some evidence for the presence of interference between beneficial mutations was found, although only relatively few sweeps were detected by the CLR test and only one closely linked pair of putative selected sites. On the other hand, in the remaining part of *Agouti* two regions of strong differentiation between OFF and ON populations were identified by the HapFLK statistic [19]. In Region 2, we found two clusters of closely linked sites that show large frequency differences between the ON and South-OFF variants and high $F_{ST}$ values at these sites. Tajima’s *D* around these two clusters was the highest in the whole *Agouti* region. Interestingly, none of the variants in the two clusters is very close to fixation in the ON population (as in the *Drosophila* example). This may be a consequence of interference (i.e., increased fixation times, see Section 2) and thus in agreement with our model: if these variants were to evolve independently, their sojourn times to fixation should be rather short (around 2300 generations or 550 to 1100 years for selection coefficients as low as 0.01; see the formulas for the fixation time in this section).

On the other hand, we cannot rule out alternative population genetic mechanisms affecting this observation, in particular explanations based on gene flow. Indeed, in both examples we discussed population structure has played an important role in the interpretation of the data. It should therefore be considered in future studies of interference between competing beneficial mutations.

## 6. Conclusions

For two closely linked beneficial mutations A and B we derived formulas for the fixation time of the recombinant haplotype AB, assuming that B has a higher fitness than A and arises before A fixes. Interference between the two mutations may lead to a substantially increased fixation time of AB in chromosomal regions of normal recombination rates. We explain how evidence for interference between competing beneficial mutations may be detected in DNA polymorphism data using estimates of selection coefficients and frequencies of selected substitutions from studies of selective sweeps. We discuss two cases in which selected substitutions (selected sweeps) occur in close proximity in the genome. One of them was found at the *polyhomeotic* locus of a European population of *D. melanogaster*. Although five closely linked selected substitutions were detected, a low level of Tajima’s D around these sites indicates that these mutations did not evolve independently. Thus, this example is a valuable negative result. A second insightful example is provided by the *Agouti* locus of deer mice. In this case, two closely linked selective sweeps were detected, but Tajima’s D around them was not elevated. Thus, they do not show signatures of interference either. On the other hand, evidence for interference is suggested by an analysis of genetic differentiation between populations on and off the Sand Hills: in a region of Intron 1, the highest peak in the HapFLK profile co-localizes with strong linkage disequilibrium and high Tajima’s D.

## Figures and Tables

**Table 1:** Simulation data of Chevin et al. [11] and predictions of fixation times.

*X*_2_(0)	$\pi_{l}$	$\pi_{m}$	$\pi_{r}$	D	$T^{r}$	$T^{a}$	T
0.007	0.333	0.491	0.191	0.503	162.7	104.8	162.3
0.024	0.311	0.473	0.201	0.535	151.6	103.3	150.4
0.077	0.276	0.42	0.198	0.468	141.8	100.2	138.6
0.224	0.244	0.323	0.192	0.019	133.1	97.1	125
0.5	0.184	0.184	0.168	−0.621	127.1	97	112.1

The first five columns show the simulation data: $X_{2}\left(0\right),\;$ the frequency of the first mutation when the second mutation is introduced; $\pi_{l},$ relative genetic diversity at a neutral locus between loci 1 and 2 near locus 1 (here ’relative’ refers to expected diversity under neutrality); $\pi_{m},$ relative genetic diversity at neutral locus in the middle between loci 1 and 2; $\pi_{r},$ relative genetic diversity at a neutral locus between loci 1 and 2 near locus 2; D, Tajima’s [17] measure of the deviation of the level of variation from neutrality at the locus in the middle. $T^{r}\;$ is the fixation time in the repulsion case from Equation (7) measured in generations, and $T^{a}\;$ is obtained for the association case by solving Equation (13) numerically. T is the fixation time averaged over both the repulsion and association cases using Equation (15). The parameter values are: $N=20,000,\;\delta =\frac{1}{N},\;s_{2}=0.1,\;s_{3}=0.2,\;and\;r=0.005;$ furthermore, $X_{1}\left(0\right)=0\;$ and $X_{3}\left(0\right)=\frac{1}{N}$ in the repulsion case, and $X_{1}\left(0\right)=\frac{1}{N}\;$ and $X_{3}\left(0\right)=\;0$ in the association case. Note that the value of $T^{r}\;$ for $X_{2}\left(0\right)=0.024\;$ in [12] is corrected here.

## Data Availability

The simulation data analyzed are contained in Table 1. A link to the publicly archived deer mice data re-analyzed is also provided.

## Appendix A

*Relationship of our Results to those of Bossert and Pfaffelhuber* [3].

Based on Equation (2.6) of Bossert and Pfaffelhuber [3], we can write (in our notation) that the time between the introduction of the second mutation into the population and its establishment is approximately

(A1) $$t_{2}-t_{1}=\frac{1}{s_{3}-s_{2}}\left(1-\frac{s_{3}\psi }{s_{2}}\right)ln\left(N\right)\;,$$

where $\psi =-\frac{ln(X_{2}(0)}{ln(N)}$. Inserting ψ into Equation (A1) leads immediately to the expression $\frac{s_{3}ln\left(X_{2}\left(0\right)\right)}{s_{2}\left(s_{3}-s_{2}\right)}-\frac{ln\left(X_{3}\left(0\right)\right)}{s_{3}-s_{2}}$ in Equation (6) and hence to the corresponding expression in Equation (7), in which population size *N* is introduced into the initial frequencies $X_{2}\left(0\right)$ and $X_{3}(0)$.
